# Quantification of syntrophic acetate-oxidizing microbial communities in biogas processes

**DOI:** 10.1111/j.1758-2229.2011.00249.x

**Published:** 2011-08

**Authors:** Maria Westerholm, Jan Dolfing, Angela Sherry, Neil D Gray, Ian M Head, Anna Schnürer

**Affiliations:** 1 Department of Microbiology, Swedish University of Agricultural SciencesSE-750 07 Uppsala, Sweden; 2 School of Civil Engineering and Geosciences, Newcastle UniversityNewcastle upon Tyne NE1 7RU, UK

## Abstract

Changes in communities of syntrophic acetate-oxidizing bacteria (SAOB) and methanogens caused by elevated ammonia levels were quantified in laboratory-scale methanogenic biogas reactors operating at moderate temperature (37°C) using quantitative polymerase chain reaction (qPCR). The experimental reactor was subjected to gradually increasing ammonia levels (0.8–6.9 g NH_4_^+^-N l^−1^), whereas the level of ammonia in the control reactor was kept low (0.65–0.90 g NH_4_^+^-N l^−1^) during the entire period of operation (660 days). Acetate oxidation in the experimental reactor, indicated by increased production of ^14^CO_2_ from acetate labelled in the methyl carbon, occurred when ammonia levels reached 5.5 and 6.9 g NH_4_^+^-N l^−1^. Syntrophic acetate oxidizers targeted by newly designed qPCR primers were *Thermacetogenium phaeum*, *Clostridium ultunense*, *Syntrophaceticus schinkii* and *Tepidanaerobacter acetatoxydans*. The results showed a significant increase in abundance of all these bacteria except *T. phaeum* in the ammonia-stressed reactor, coincident with the shift to syntrophic acetate oxidation. As the abundance of the bacteria increased, a simultaneous decrease was observed in the abundance of aceticlastic methanogens from the families *Methanosaetaceae* and *Methanosarcinaceae*. qPCR analyses of sludge from two additional high ammonia processes, in which methane production from acetate proceeded through syntrophic acetate oxidation (reactor SB) or through aceticlastic degradation (reactor DVX), demonstrated that SAOB were significantly more abundant in the SB reactor than in the DVX reactor.

## Introduction

Methane formation from acetate can proceed through two different mechanisms. The most commonly described involves aceticlastic methanogens that perform acetate cleavage for methane production. The second mechanism proceeds through syntrophic acetate oxidation (Zinder and Koch, 1984). This pathway entails fermentation of acetate to hydrogen and carbon dioxide by syntrophic acetate-oxidizing bacteria (SAOB). Hydrogen utilizing methanogens then reduce carbon dioxide to methane. High ammonia levels, formed during the anaerobic degradation of protein-rich material, have been shown to be one important factor regulating the shift from aceticlastic methanogenesis to syntrophic acetate oxidation in mesophilic biogas processes (Schnürer *et al*., 1999; Schnürer and Nordberg, 2008). The shift is probably a consequence of the inhibitive effect of ammonia on the activity of the aceticlastic methanogens (Koster and Lettinga, 1984; Sprott and Patel, 1986). The concentration of acetate, dilution rate and presence of the aceticlastic *Methanosaetaceae* are other factors suggested to have an impact on the development of syntrophic acetate oxidation (Petersen and Ahring, 1991; Ahring *et al*., 1993; Shigematsu *et al*., 2004; Karakashev *et al*., 2006).

So far a restricted number of SAOB have been isolated and characterized, namely the mesophilic bacteria *Clostridium ultunense* (Schnürer *et al*., 1996; 1997) and *Syntrophaceticus schinkii* (Westerholm *et al*., 2010), the thermotolerant *Tepidanaerobacter acetatoxydans* (Westerholm *et al*., 2011), and the thermophilic bacteria *Thermacetogenium phaeum* (Hattori *et al*., 2000; 2005) and *Thermotoga lettingae* (Balk *et al*., 2002). Initially, a thermophilic bacterium (Lee and Zinder, 1988) named *Reversibacter* was described, but unfortunately this bacterium was lost before its phylogenetic position could be established.

Information about syntrophic acetate oxidation, the organisms involved, and their role in the methanogenic environment is currently limited. However, greater understanding of microbial dynamics in response to inhibitory compounds, such as ammonia, should facilitate further development and also optimization of the anaerobic treatment process. In the present study, primers targeting 16S rRNA gene sequences of known SAOB were designed. Quantitative real-time polymerase chainreaction (qPCR) analyses were then performed in order to determine changes in SAOB and methanogenic communities caused by elevated ammonia concentrations. Two mesophilic biogas reactors were included in the analysis. In one (experimental) reactor a shift from aceticlastic acetate degradation to syntrophic acetate oxidation had been established previously, while in the second (control) reactor aceticlastic methanogenesis was the main pathway for methane formation (Schnürer and Nordberg, 2008). Two high ammonia processes, in which methane production from acetate proceeded through syntrophic acetate oxidation (reactor SB) or through aceticlastic degradation (reactor DVX), were also included in the investigation.

## Results and discussion

### Pathway for acetate degradation in the biogas reactors

In the control reactor, acetate degradation was primarily through aceticlastic methanogenesis throughout the operating period. In the experimental reactor, which was subjected to gradually increasing ammonia levels, a shift from aceticlastic acetate degradation to syntrophic acetate oxidation was established between 225 and 442 days of operation, when the ammonia level reached 5.5 and 6.9 g NH_4_^+^-N l^−1^ (Fig. S1). Labelling analysis with [2-^14^C]-acetate demonstrated occurrence of syntrophic acetate oxidation in reactor SB (Schnürer and Nordberg, 2008; Ek *et al*., 2010), while in reactor DVX the analysis indicated that aceticlastic methanogenesis was the main pathway for acetate degradation (Fig. S1). The dominance of aceticlastic methanogenesis in reactor DVX was somewhat unexpected, as the ammonia concentration in this reactor exceeded the levels previously shown to cause development of syntrophic acetate oxidation (Schnürer *et al*., 1999; Schnürer and Nordberg, 2008). Parameters other than ammonia [e.g. substrate change, increased loading rate and decreased hydraulic retention time (HRT)] apparently had an impact on the mechanism developed for methane formation in reactor DVX.

### Primer specificity and detection of SAOB in samples with conventional PCR

Specific primer sets for detection of 16S ribosomal RNA (rRNA) genes of *C. ultunense*, *S. schinkii*, *T. acetatoxydans* and *T. phaeum* (Table 1) generated single PCR products from genomic DNA of the corresponding species. Furthermore, in PCR amplification with primer sets targeting *C. ultunense*, *S. schinkii* and *T. acetatoxydans*, products of the predicted length were generated from template DNA extracted from the experimental reactor on day 442 and day 642 of operation. From all other samples (control and experimental reactor) the quantity of amplified DNA was below the detection limit for visualization using ethidium bromide staining. Furthermore, PCR analysis with the primer set targeting the 16S rRNA gene of the thermophile *T. phaeum* did not generate any visible product from any reactor sample. Analysis of DNA extracted from samples from reactors SB or DVX only gave a positive result with the primer set targeting the 16S rRNA gene of *S. schinkii*. The primers all showed high specificity, as PCR products generated from all reactor samples were sequenced and shown to be identical (100% identity over 171, 237 and 127 bp respectively) to the sequences retrieved from pure cultures of the corresponding bacteria. Furthermore, all primer sets showed high specificity to the corresponding SAOB in an evaluation against the GenBank database using BLAST.

**Table 1:** Primer sets and PCR programs used in the investigation

Primer^a^	Target species or group	Sequence (5′→3′)^b^	Position in target species^c^	T_m_ (°C)	Amplicon size (bp)
Cultf^e^	*Clostridium ultunense*	CCT TCG GGT GGA ATG ATA AA	56–76	57	127
Cultr^e^		TCA TGC GAT TGC TAA GTT TCA	162–183		
THACf^d^	*Syntrophaceticus schinkii*	ATC AAC CCC ATC TGT GCC	802–820	61	171
THACr^d^		CAG AAT TCG CAG GAT GTC	955–973		
Tpf^d^	*Tepidanaerobacter acetatoxydans*	AGG TAG TAG AGA GCG GAA AC	963–983	63	237
Tpr^d^		TGT CGC CCA GAC CAT AAA	1182–1200		
Thf^e^	*Thermacetogenium phaeum*	GGG TGG TGT GAA GCC ATC	795–813	68	175
Thr^e^		AGG TCC GCA GAG ATG TCA AG	970–990		
Tbf^f^	Total bacteria	GTG ITG CAI GGI IGT CGT CA	1048–1068	61	323
Tbr^f^		ACG TCI TCC ICI CCT TCC TC	1371–1391		
Mscf^g^	*Methanosarcinaceae*	GAA ACC GYG ATA AGG GGA	380–397	60	408
Mscr^g^		TAG CGA RCA TCG TTT ACG	811–828		
MMBf^g^	*Methanomicrobiales*	ATC GRT ACG GGT TGT GGG	282–299	66	506
MMBr^g^		CAC CTA ACG CRC ATH GTT TAC	812–832		
Mstf^g^	*Methanosaetaceae*	TAA TCC TYG ARG GAC CAC CA	702–721	61	164
Mstr^g^		CCT ACG GCA CCR ACM AC	812–832		
pAf^h^	Bacteria	AGA GTT TGA TCC TGG CTC AG	8–28	55	1534
pHr^h^		AAG GAG GTG ATC CAG CCG CA	1542–1522		
Arch46f^i^	*Archaea*	YTA AGC CAT GCR AGT	46–61	40	971
Arch1017r^j^		GGC CAT GCA CCW CCT CTC	1017–999		

a f, forward; r, reverse primer.

b I, inosine.

c 16S rRNA gene sequence.

d Designed by Dr Neil Gray, School of Civil Engineering and Geosciences; Newcastle University.

e Designed by Stefan Roos and Maria Westerholm, Department of Microbiology, Swedish University of Agricultural Sciences, Uppsala, Sweden.

f (Maeda *et al*., 2003).

g (Yu *et al*., 2005).

h (Edwards *et al*., 1989).

i (Øvreås *et al*., 1997).

j (Barns *et al*., 1994).

Primer sets targeting the 16S rRNA genes of *S. schinkii* and *T. acetatoxydans* were designed with Primrose version 2.1.7 (Ashelford *et al*., 2002) and for amplification of the 16S rRNA genes of *C. ultunense* and *T. phaeum*, primers were designed with Primer3, version 0.4.0 (Rozen and Skaletsky, 2000). The primer specificity was evaluated against the GenBank database using blast (Altschul *et al*., 1990). PCR amplifications were conducted using a 25 µl mixture including 5 µl of 10x NH_4_ buffer (Bioline, London, UK), 1.5 µl of 50 mM MgCl_2_, 1 µl of forward and reverse primer (10 µM), 1 µl of dNTPs (10 mM each), 0.2 µl of *Taq* DNA polymerase (Bioline, London, UK), 14.3 µl of sterile water and 1 µl of template DNA in each reaction. Alternatively Ready-To-Go PCR beads (GE Healthcare Buckinghamshire, UK), containing 25 pmol of each primer per 25 µl PCR reaction, were used. The PCR program consisted of: 95°C for 3 min, 30 cycles of 95°C for 1 min, annealing for 1 min at temperature shown above, and 72°C for 1 min, followed by 10 min at 72°C.

### Real-time PCR quantification of SAOB and methanogens

All standard curves for the quantitative PCR analyses, constructed as described in Table S1, had a linear correlation coefficient (*r*^2^) ranging between 0.985 and 0.999, and the calculated qPCR efficiency of the reactions varied between 86.2% and 108%.

**Fig. 1:**
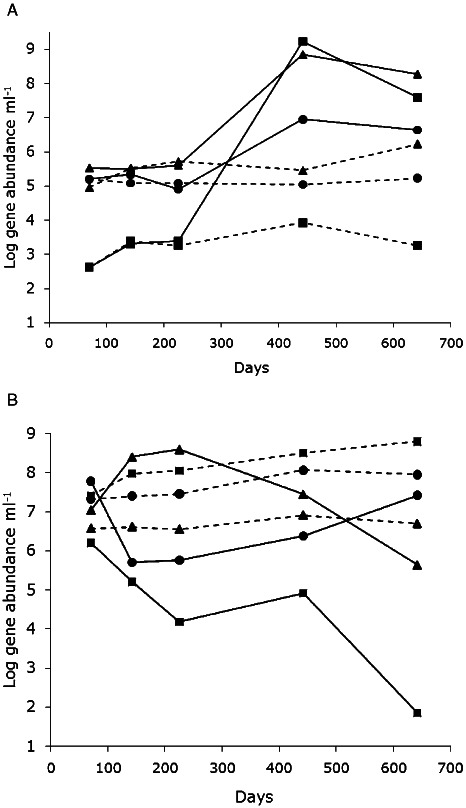
(A) Abundance of syntrophic acetate-oxidizing bacteria in the control reactor (- - -) and the experimental reactor (—), as determined by qPCR analysis of 16S rRNA genes. *C. ultunense*•; *S. schinkii*▴; *T. acetatoxydans*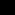. (B) Abundance of methanogens in the control reactor (- - -) and the experimental reactor (—) as determined by qPCR analysis of 16S rRNA genes. *Methanomicrobiales*•; *Methanosarcinaceae*▴; *Methanosaetaceae*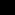. Genomic DNA was extracted from three replicate samples (0.3 ml each) from each reactor and sampling point, using the FastDNA Spin kit for soil (Qbiogene, Illkrich, France). The triplicate DNA samples were pooled and the qPCR was performed with a BioRad iCycler (Hercules, CA). Each reaction contained 3 μl DNA template, 1 μl of each primer (10 pmol μl^−1^), 5 μl of sterile water, 10 μl iQ Supermix PCR reagent (BioRad, Hercules, CA), and SYBR-Green I as the fluorescent DNA intercalating agent (0.2 μl of 100x concentrate, Invitrogen, UK). In qPCR analysis of the methanogenic communities the temperature cycle consisted of: 95°C for 7 min; 55 cycles of 95°C for 40 s; annealing at specific temperatures (Table S1) for 1 min; and 72°C for 40 s. qPCR analysis of SAOB was performed applying the following conditions: 7 min at 95°C; 40 cycles of 95°C for 30 s; annealing at specific temperatures (Table S1) for 1 min; and 72°C for 30 s. At the end of each qPCR assay, a temperature melt curve was performed to verify reaction quality (55–95°C, ΔT = 0.1°C s^−1^). Logarithmic values of the concentration of the16S rRNA gene were plotted against the threshold cycle (C_t_) number and used for estimation of gene abundance in the unknown samples.

The qPCR analyses showed a distinct increase in *C. ultunense*, *S. schinkii* and *T. acetatoxydans* in the experimental reactor when the ammonia level increased above 3.3 g NH_4_^+^-N l^−1^ (Fig. 1A). The increase was confirmed by an additional assay with triplicate DNA samples from the experimental reactor on day 225 and day 442, which demonstrated a significant increase (un-paired *t*-test, P < 0.05) in *C. ultunense* from 4.1 ± 1.2 × 10^5^ to 2.3 ± 0.9 × 10^7^ gene abundance ml^−1^, in *S. schinkii* from 6.3 ± 1.4 × 10^6^ to 6.8 ± 2.1 × 10^9^ gene abundance ml^−1^, and in *T. acetatoxydans* from 4.7 ± 2.4 × 10^5^ to 5.7 ± 0.4 × 10^10^ gene abundance ml^−1^. In parallel, a decrease in the abundance of acetate utilizing methanogens from the family *Methanosarcinaceae* occurred from day 225 onwards, when the ammonia concentration exceeded 3.3 g NH_4_^+^-N l^−1^. However, the abundance of the acetate utilizing *Methanosaetaceae* declined after only 70 days of operation (Fig. 1B). Hydrogenotrophic methanogens of the order *Methanomicrobiales* initially decreased in abundance between days 70 and 142, but subsequently increased to around their initial abundance by day 642. It is possible that certain members of the *Methanomicrobiales* declined initially due to ammonia inhibition or pH change and subsequently (> 142 days) ammonia-tolerant members of the *Methanomicrobiales* were favoured as the ammonia concentration increased. The observed decrease in aceticlastic methanogens and increase in hydrogenotrophic methanogens in response to increasing ammonia levels, most likely caused by a comparatively higher tolerance of *Methanomicrobiales* to ammonia (Koster and Lettinga, 1984; Sprott and Patel, 1986), have been reported at population level previously (Angenent *et al*., 2002). However, the present study represents the first detailed analysis of changes in both the population of methanogens and SAOB in response to increasing ammonia concentration.

In the control reactor, methanogen and SAOB abundance remained stable throughout the 642 days of operation (Fig. 1). Total bacterial abundance in the control reactor and experimental reactor was stable (4.9 ± 1.8 × 10^10^ and 3.7 ± 2.2 × 10^10^ gene abundance ml^−1^ respectively) throughout the operating period. These results supported the presumption that the changes in the microbial communities in the experimental reactor were a consequence of increased ammonia concentration.

16S rRNA genes from *T. phaeum* were not detected in any of the reactors. This was not surprising, as the temperature range of this thermophilic bacterium is 40–65°C, with an optimum around 58°C. The conditions in the reactors, operating at 37°C, were therefore unfavourable for *T. phaeum*.

In previous studies, *C. ultunense*, *S. schinkii* and *T. acetatoxydans* proved capable of withstanding rather high levels of ammonium chloride (∼ 8 g NH_4_^+^-N l^−1^) at neutral pH (Schnürer *et al*., 1996; Westerholm *et al*., 2010; 2011), an ammonium level that has strong inhibitory effects on aceticlastic methanogens from the families *Methanosarcinaceae* and *Methanosaetaceae* (Sprott and Patel, 1986; Hajarnis and Ranade, 1993). The ammonia tolerance of these syntrophic acetate-oxidizers probably gives them a competitive advantage in ammonia-stressed systems. These bacteria, in association with ammonia-tolerant hydrogenotrophic methanogens, may consequently adopt the role of dominant acetate consumers in environments where ammonia restrains aceticlastic methanogenic activity.

**Fig. 2:**
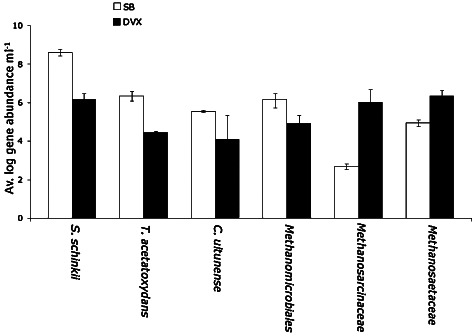
Abundance of syntrophic acetate oxidizing bacteria and methanogens in reactors SB and DVX. Acetate degradation proceeded through syntrophic acetate oxidation in reactor SB and via aceticlastic methanogenesis in reactor DVX. SB was a large-scale reactor operating with an average HRT of 56 days and was fed with slaughterhouse waste as main substrate (Ek *et al.*, 2010). At the time of sampling the concentrations of volatile fatty acids (VFA) and ammonia-nitrogen in the process were 2.3 g l^−1^ and 5.3 g NH_4_^+^-N l^−1^, respectively. DVX was a laboratory-scale reactor that was inoculated with sludge from the SB reactor. The process was fed with distiller's waste and operated with an average HRT of ∼40 days at approximately pH 7.8, 1.6 g VFA l^−1^ and 7.8 g NH_4_^+^-N l^−1^. The OLR of DVX was initially 4 g VS l^−1^ day^−1^ and was then gradually increased and had reached 6 g VS l^−1^ day^−1^ when sampled. After ∼330 days of operation, high concentrations of VFA (4-5 g l^−1^) had accumulated in the process and the pH had started to decrease. Triplicate samples from reactors SB and DVX, taken on a single sampling occasion, were analyzed separately and the qPCR analysis was conducted as described in Fig. 1.

In reactor SB, *C. ultunense*, *S. schinkii* and *T. acetatoxydans* were present at significantly (*t*-test, P < 0.05) greater abundance than in reactor DVX (Fig. 2). The total bacterial gene abundance in reactor SB (1.0 ± 0.4 × 10^11^ ml^−1^) was also slightly higher than in reactor DVX (3.2 ± 0.8 × 10^10^ ml^−1^). The comparatively low abundance of acetate oxidizers in reactor DVX agreed with the labelling analysis, demonstrating dominance of aceticlastic methanogenesis in this reactor. In contrast, there was no significant difference (*t*-test, P > 0.05) in mean gene abundance of *Methanosarcinaceae* or *Methanomicrobiales* between reactors DVX and SB, and the *Methanosaetaceae* abundance was even significantly lower in reactor DVX. The high abundance of *Methanosaetaceae* in reactor SB was unexpected and contradicted results reported by Karakashev and colleagues (2006), showing that acetate oxidation is the dominant pathway only in the absence of *Methanosaetaceae*. The relatively high abundance of *Methanosarcinaceae* and *Methanosaetaceae* in reactor DVX is also noteworthy, indicating occurrence of ammonia-tolerant aceticlastic methanogens in this reactor operating at a high ammonia concentration. However, the accumulation of VFA and the decline in pH demonstrated the instability of aceticlastic methanogenesis in the conditions under which reactor DVX was operated, thereby reflecting the importance of SAOB for the maintenance of process stability in methanogenic systems with high ammonia concentrations.

## Supporting information

**Fig. S1.** Degree of acetate oxidation measuredas ^14^CO_2_/^14^CH_4_ over theoperational period of 642 days in the control and experimentalreactor, described by Schnürer and Nordberg (2008). Theconcentration of ammonia-nitrogen (g NH_4_^+^-Nl^−1^) is stated above the bars. Samples withdrawnat a single sampling point from two high ammonia processes,designated SB and DVX were also included in this investigation.Samples for DNA extraction and [2-^14^C]-acetate traceranalysis were withdrawn from the SB reactor after 12 years ofoperation and from DVX reactor after 270 days of operation. Degreeof ^14^CO_2_/^14^CH_4_ in DVXand SB are mean values of two measurements. All processes operatedat moderate temperature (37°C).

**Table S1.** Construction of DNA standards for quantification.
