# Supplementary material for: Quantification of syntrophic acetate-oxidizing microbial communities in biogas processes
Source: Environ Microbiol Rep. 2011 Aug;3(4):500–5. doi: 10.1111/j.1758-2229.2011.00249.x (PMC3659410; doi:10.1111/j.1758-2229.2011.00249.x)
Supplement: Supplementary file 1 [file emi40003-0500-SD1.doc]

**Supplementary Fig. S1.**


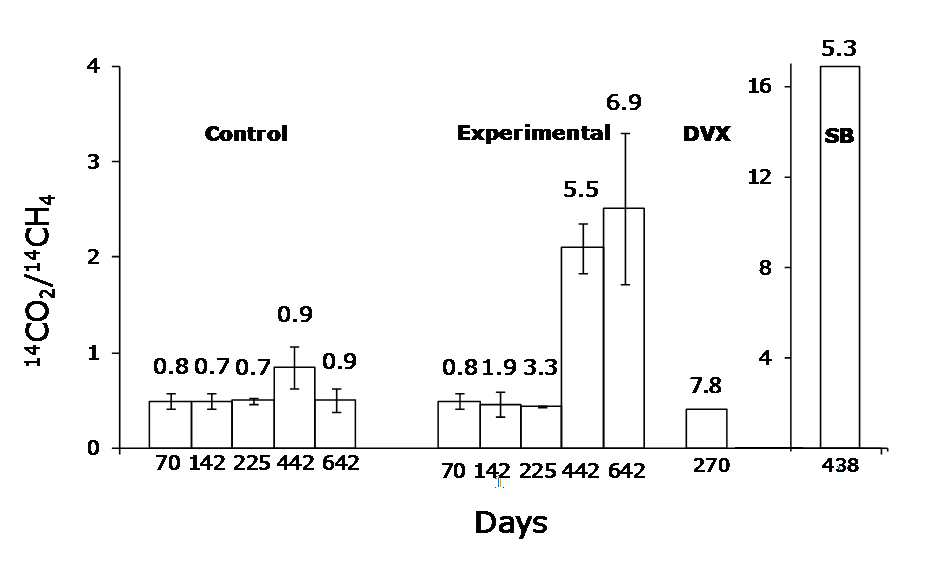


**Fig. 1.** Degree of acetate oxidation measured as ^14^CO_2_/^14^CH_4_ over the operational period of 642 days in the control and experimental reactor, described by Schnürer and Nordberg (2008). The concentration of ammonia-nitrogen (g NH_4_^+^-N l^-1^) is stated above the bars. Samples withdrawn at a single sampling point from two high ammonia processes, designated SB and DVX were also included in this investigation. Samples for DNA extraction and [2-^14^C]-acetate tracer analysis were withdrawn from the SB reactor after 12 years of operation and from DVX reactor after 270 days of operation. Degree of ^14^CO_2_/^14^CH_4_ in DVX and SB are mean values of two measurements. All processes operated at moderate temperature (37°C).

**Reference in Fig. S1**

Schnürer, A., and Nordberg, A. (2008) Ammonia, a selective agent for methane production by syntrophic acetate oxidation at mesophilic temperature. *Water Sci Technol* **57**: 735-740.
