# Supplementary material for: Quantification of syntrophic acetate-oxidizing microbial communities in biogas processes
Source: Environ Microbiol Rep. 2011 Aug;3(4):500–5. doi: 10.1111/j.1758-2229.2011.00249.x (PMC3659410; doi:10.1111/j.1758-2229.2011.00249.x)
Supplement: Supplementary file 2 [file emi40003-0500-SD2.doc]

**Supplementary Table S1.** Construction of DNA standards for quantification

| Primer set | Species/sample used for extraction of genomic DNA | Procedure for DNA standard preparation |  | Primers used for amplification of partial 16S rRNA gene^a^ |
| --- | --- | --- | --- | --- |
| Cult | *Clostridium ultunense* sp. Esp  JCM 16670 | PCR |  | 16ss  D1492r^b^ |
|
| THAC | *Syntrophaceticus schinkii*  JCM 16669 | Cloning |  | THACf  THACr |
|
| Tp | *Tepidanaerobacter acetatoxydans* DSM 21804 | PCR |  | pA  pH^c^ |
|
| Th | *Thermacetogenium phaeum*  DSM 12270 | PCR |  | 16ss  D1492r^b^ |
|
| Tb | *Nitrospira* *moscoviensis*  DSM 10035 | Cloning |  | Tbf  Tbr |
|
| Msc | environmental samples | Cloning |  | Arch46f  Arch1017r |
|
| MMB | environmental samples | Cloning |  | Arch46f  Arch1017r |
|
| Mst | *Methanosaeta concilii* DSM 2139 | Cloning |  | Mstf  Mstr |
|

^a^ PCR program and primer sequence are specified in Table 1.

^b^ PCR program and primer sequence as described by Westerholm *et al*. (2010).

^c^ The PCR program given in Table 1 was modified with a final extension step of 10 min at 95°C.

Two different methods were used for the preparation of DNA standards due to different standard procedures at different laboratories. In the PCR procedure DNA standards were constructed using PCR amplified partial 16S rRNA genes from genomic DNA of bacterial pure cultures. PCR products were gel-purified (QIAquick gel extraction kit; Qiagen, Hilden, Germany). In the cloning procedure, 16S rRNA genes amplified from genomic DNA were gel purified, cloned into the pCR4-TOPO vector (Invitrogen, Carlsbad, CA) and then transformed into component *E. coli* cells, in accordance to the manufacturer’s instructions. Plasmids were extracted from an overnight culture using the QIAprep spin miniprep kit (Qiagen, Hilden, Germany). For construction of DNA standards for *Methanomicrobiales* and *Methanosarcinaceae* the clones were sequenced to identify relevant methanogen sequences. Clones containing the correct insert were PCR-amplified using the vector-specific primers pUCF and pUCR, followed by gel purification of the product. DNA concentration in the purified standards was determined with a NanoVue spectrophotometer (GE healthcare, Buckinghamshire, UK), and standards were prepared to a concentration of 10^9^ molecules µl^-1^ according to Equation 1. DNA standards were 10-fold serially diluted and were run in duplicate or triplicate alongside each qPCR assay. To assess levels of background contamination, triplicate wells containing reaction mix without template DNA were included in each assay. All samples were analyzed for inhibitory effects on qPCR performance as described previously (Wessén *et al.*, 2010).

Equation1:

16S rRNA genes (molecules µl^-1^) =

(where MW = 16S rRNA gene amplicon size [bp] × 660 Da).

**References in Table**

Wessén, E., Hallin, S., and Philippot, L. (2010) Differential responses of bacterial and archaeal groups at high taxonomical ranks to soil management. *Soil Biol Biochem* **42**: 1759-1765.

Westerholm, M., Roos, S., and Schnürer, A. (2010) *Syntrophaceticus schinkii* gen. nov., sp. nov., an anaerobic, syntrophic acetate-oxidizing bacterium isolated from a mesophilic anaerobic filter. *FEMS Microbiol Lett* **309**: 100-104.
